# Brief Educational Workshops in Secondary Schools Trial (BESST): protocol for a school-based cluster randomised controlled trial of open-access psychological workshop programme for 16–18-year-olds

**DOI:** 10.1186/s13063-022-06830-8

**Published:** 2022-11-09

**Authors:** Stephen Lisk, Ben Carter, Kirsty James, Paul Stallard, Jessica Deighton, Jynna Yarrum, Peter Fonagy, Crispin Day, Sarah Byford, James Shearer, Timothy Weaver, Irene Sclare, Claire Evans, Maria Farrelly, Pin-Cheng Ho, June Brown, BESST Study Collaborators

**Affiliations:** 1 King’s College London, London, UK; 2 Department of Biostatistics and Health Informatics, Institute of Psychiatry, Psychology & Neuroscience, King’s College London, London, UK; 3 King’s Clinical Trials Unit, Institute of Psychiatry, Psychology & Neuroscience, King’s College London, London, UK; 4 University of Bath, Bath, UK; 5 Anna Freud National Centre for Children and Families, London, UK; 6 University of Northampton, Northampton, UK; 7 Department of Clinical Educational and Health Psychology, University College London, London, UK; 8 Middlesex University, London, UK; 9 South London and Maudsley NHS Foundation Trust, London, UK

**Keywords:** Adolescents, Anxiety, CBT, Depression, Schools

## Abstract

Anxiety and depression are increasingly prevalent in adolescents, often causing daily distress and negative long-term outcomes. Despite the significant and growing burden, less than 25% of those with probable diagnosis of anxiety and depression are receiving help in England. Significant barriers to help-seeking exist in this population, with a scarcity of easily accessible, effective, and cost-effective interventions tailored specially for this age group. One intervention that has been shown to be feasible to deliver and with the promise of reducing stress in this age group is a school-based stress workshop programme for 16–18-year-olds (herein called DISCOVER). The next step is to rigorously assess the effectiveness, and cost-effectiveness, of the DISCOVER intervention in a fully powered cluster randomised controlled trial (cRCT). If found to be clinically and cost-effective, DISCOVER could be scaled up as a service model UK-wide and have a meaningful impact on the mental health of adolescents across the country.

**Trial registration:** ISRCTN registry ISRCTN90912799. Registered with ISRCTN 28 May 2020.

**Supplementary Information:**

The online version contains supplementary material available at 10.1186/s13063-022-06830-8.

## Administrative information

Note: the numbers in curly brackets in this protocol refer to SPIRIT checklist item numbers. The order of the items has been modified to group similar items (see http://www.equator-network.org/reporting-guidelines/spirit-2013-statement-defining-standard-protocol-items-for-clinical-trials/).

**Table:** 

Title {1}	Early intervention for depression and anxiety in 16–18-year-olds: a multi-centre, cluster randomised control trial of a self-referral psychological stress workshop programme in schools (BESST study—Brief Educational workshops in Secondary Schools Trial)
Trial registration {2a and 2b}.	ISRCTN90912799 (registered with ISRCTN 28 May 2020).
Protocol version {3}	Version 1.2.2—03/02/22
Funding {4}	This project is funded by the National Institute for Health Research (NIHR) Health Technology Assessment (HTA) Programme (project reference NIHR127951).
Author details {5a}	a. King’s College London, London, UK b. Department of Biostatistics and Health Informatics, Institute of Psychiatry, Psychology & Neuroscience, King’s College London, London, UK c. King’s Clinical Trials Unit, Institute of Psychiatry, Psychology & Neuroscience, King’s College London, London, UK d. University of Bath, Bath, UK e. Anna Freud National Centre for Children and Families, London, UK f. University of Northampton, Northampton, UK g. Department of Clinical Educational and Health Psychology, University College London, London, UK h. Middlesex University, London, UK i. South London and Maudsley NHS Foundation Trust, London, UK
Name and contact information for the trial sponsor {5b}	King’s College London, Room 1.8 Hodgkin Building Guy's Campus, King's College London. Telephone: 02078486960
Role of sponsor {5c}	The sponsor has no role in study design, data collection, analysis, data interpretation or writing of the research reports. There are no financial or other competing interests for the principal investigators for the overall trial and each study site.

## Introduction

### Background and rationale {6a}

More than half of adult mental disorders have first onset before the age of 15 years, and almost three-quarters by the age of 18 [1, 2]. Emotional disorders of anxiety and depression are especially common in the adolescent years, causing marked distress and daily interference for 5% of teenagers at any given time [3, 4]. Anxious and depressed young people are more likely to suffer from poor social, educational, and occupational outcomes [5–7]. They are also vulnerable to substance abuse, early sexual activity, and self-harm [8, 9]. Even sub-threshold emotional symptoms, which affect up to one-third of adolescents [10], increase the risk for long-term functional impairment and suicidal behaviours [11, 12].

Diagnosis of an anxiety disorder in childhood and adolescence is the most common risk factor for anxiety and depression in adulthood [2] which may not be surprising given that the majority of childhood and adolescent anxiety disorders remain untreated [13]. In fact, less than one-quarter of anxious and depressed youth are in contact with specialist child and adolescent mental health services (CAMHS) in the UK [14]. A significant portion of young people choose not to disclose problems due to concerns about stigma and confidentiality [15, 16]. Factors such as inconvenient appointment times, transportation difficulties, long waiting lists, and high thresholds for specialist referral have also been identified as barriers to care [17, 18]. Furthermore, even when young people do access mental health clinics, there is limited provision of cognitive-behavioural therapy (CBT) and other evidence-based psychological therapies [19].

Given these significant barriers to treatment access and the continuous rise in adolescent mental health issues, identification of effective and readily accessible resources and interventions is a public health priority. These also need to be scalable. One approach to improve accessibility is to use schools as a location for mental health services and care delivery [20, 21]. In fact, given the rise in adolescent mental health issues [22], researchers have begun to develop school-based mental health programmes to combat stress, anxiety, and depressive symptoms in adolescents.

A recent systematic review and meta-analysis conducted by Feiss and colleagues [23] identified 42 studies in the US evaluating school-based stress, anxiety, and depression interventions for adolescents. The majority of studies (35 of 42 identified) used ‘traditional’ (CBT-based) approaches, with most studies also utilising a group-based approach. Other approaches included meditation-based approaches and other holistic approaches. The meta-analyses provided somewhat encouraging results, reporting a modest reduction of anxiety and depression symptoms following school-based interventions focusing on those specific disorders. A slightly earlier meta-analysis performed by Werner-Seidler and colleagues [24] also found small but encouraging effects of programmes for depression and anxiety prevention, which remained at a 12-month follow-up.

The studies identified in these reviews typically reported intervention access on either a universal (provided to all students) or targeted basis (for students identified as exhibiting elevated or clinical symptoms). Both reviews reported that targeted programmes were more effective in reducing depressive symptoms than universal programmes. However, this is likely to be due to the floor effect of symptom reduction, due to a substantial number of participants in universal programmes not displaying elevated symptoms. Furthermore, whilst targeted approaches demonstrate greater effectiveness, they are also potentially disadvantaged by creating stigma around inclusion and excluding those who are at risk of developing issues but not currently exhibiting symptoms. A relatively novel option to address these issues is a self-referral model [25], in which students can refer themselves to an intervention without having to go through a screening process. This approach has the potential to reduce stigma, emphasise autonomy (valued by adolescents), and allow for resources to be utilised most cost-effectively for those who require them.

A number of universal interventions in this age group have also used a digital CBT approach. Small to moderate effects have been found for anxiety reduction [26] and medium effects amongst female participants for depression [27]. However, whilst attractive in terms of reach, the dropout rates have been unacceptably high, with only an average of 30% of participants completing these programmes [26, 27].

Importantly, the meta-analyses discussed above have demonstrated a lack of studies investigating these approaches specifically in older (16 +) adolescents. Studies included by Feiss et al. [23] were from high schools and middle schools, with a total age range of 11–18 years. Those studies identified targeting older adolescents in high schools, still had a relatively broad age range of 14–18 years. Similarly, Werner-Seidler et al. [24] identified 25 of the overall 81 school-based depression/anxiety programmes as targeting older adolescents: with the mean age for this sub-set of studies ranging from 14 to 19 years. In fact, to date, there appears to be a lack of research into school-based interventions specifically for older adolescents (16 +), with just one small trial (*N* = 21) specifically focusing on a school-based intervention for anxiety and depression in the 16 + age group [28]. The lack of findings for school-based interventions specifically for 16–18-year-olds is especially meaningful when considering that distinctive mental health needs of older adolescents do not readily fall in line with ‘downward adaptations’ of adult treatments or ‘upward adaptations’ of child treatments [29]. Indeed, evidence suggests the middle-to-late teen years to be a critical developmental period, with continued brain maturation and marked differences in sleep and coping mechanisms as well as significant social changes (e.g. increased autonomy and self-determination) compared to younger adolescents and adults [30–32]. Therefore, this vulnerable period of change likely requires specific interventions tailored for this population.

In England, there has been a major development in increasing these types of mental health resources available in educational settings. In 2017, the government set out (in its green paper ‘Transforming Children and Young People’s Mental Health Provision’) [22] to expand access to mental health care for children and young people in England. They proposed to achieve this by creating Mental Health Support Teams (MHST) to work between schools and child and adolescent mental health services (CAMHS) to provide early intervention on mental wellbeing, as well as providing on-going training and support for a Senior Mental Health Lead in schools and colleges. MHSTs consist of educational mental health practitioners (EMHPs) and/or Children’s Wellbeing Practitioners (CWPs) and are located within educational settings across England, led by a designated senior leader. EMHPs and CWPs receive 12-month training to assess and support young people with common mental health difficulties, using low-intensity CBT approaches to address mild to moderate symptoms of anxiety, depression, and behaviour difficulties. As of Spring 2022, 287 MHSTs were operational, covering over 4700 schools/colleges. The transformation plan aims to have nearly 400 MHSTs working with and in schools and colleges (and attended by almost 3 million pupils) by 2023.

Finally, the current literature demonstrates that data regarding ethnic background is often not fully reported. Whilst Feiss and colleagues [23] found race to moderate the effect of programme type, there was not sufficient data to decompose these interactions. Previous research has indicated that interventions may be less effective with those from more ethnically diverse backgrounds and racial minorities are often underserved in this area of research [33].

Whilst there are promising results from school-based interventions for adolescents, there is a lack of easily accessible and effective prevention programmes that are designed specifically for older adolescents, targeting depression and anxiety in school settings. Furthermore, students from ethnic minorities are potentially under-represented. However, this problem might need to be redressed in different ways. In an important review, Naeem [34] concluded that significant modifications are likely needed to make (high intensity) CBT accessible to non-Western cultures, with fully powered trials necessary for each of these adaptations. Whilst large trials with each population may not be realistically achievable, another approach is increasing the accessibility for ethnic minorities to current interventions, which may have a positive impact.

With these needs in mind, the DISCOVER workshop programme has been developed to (i) be clinically effective in reducing stress, (ii) offer cost-effectiveness in reducing stress, and (iii) increase uptake in harder to reach and treat groups (for example ethnic minority groups) by being easier to access (school-based and removal of screening measures) and by reducing stigma through neutral language. The content and delivery methods have been adapted from an established ‘well-being workshop’ model for working age adults [35, 36]. Key elements of the adult workshops are (i) use of evidence-based CBT materials; (ii) group delivery at community sites; (iii) brief, 1-day duration; and (iv) a self-referral pathway. These features were reviewed by a Teenage Advisory Group and refined in an initial proof-of-concept study [37]. The latest iteration of DISCOVER incorporates new, age-appropriate video material, a more interactive presentational style, and additional methods for personalisation and telephone follow-up.

A feasibility study of DISCOVER, a two-arm cluster randomised controlled trial [38], was conducted in 10 schools with 155 year 12 and year 13 students (aged at least 16) (*n* = 155). Female and ethnic minority groups accounted for 81% and 57.4% of students, respectively. DISCOVER comprises a pre-workshop goal setting session, a 1-day workshop, and 1–3 follow-up telephone calls. In this feasibility study, 72.2% attended the full-day intervention, 11.1% attended part of the day, and 17.7% did not attend the workshop. Even though under-powered, significant reductions were found in depression (*d* = 0.27) and anxiety (*d* = 0.25) at the 3-month follow-up after controlling for baseline measures and schools.

A qualitative study exploring the feasibility and acceptability of DISCOVER using semi-structured interviews indicated that DISCOVER was generally feasible and acceptable [39]. Three groups of participants were purposively sampled to include students from ethnic minority backgrounds: students who attended the workshop (*n* = 15), students who showed interest initially but decided not to participate (*n* = 9), and school staff who helped organise the programme in schools. Students reported that the workshop helped them understand stress and related management techniques. They showed a preference for the school setting, interactive activities, and individualised approach between psychologists and students. School staff reported that the workshop was in line with school values. They also expressed a desire for more information regarding the workshop for follow-up support and described some logistical barriers of delivery, like timetable and shortage of available classrooms. The main reason for students not participating was their limited time.

### Objectives {7}

The current study will accomplish the critical next phase of development and testing, in line with the MRC Framework for Complex Interventions [40], with a full UK-wide clinical trial to investigate the effectiveness of DISCOVER.

The primary objective is:

1. To investigate the clinical effectiveness of DISCOVER on symptoms of depression in 16–18-year-olds over 6 months-

Secondary objectives are:

1. To determine the feasibility of running a UK-wide confirmatory trial of the DISCOVER intervention
2. To assess the cost-effectiveness of DISCOVER compared to control treatment in terms of quality-adjusted life years (QALYs)
3. To assess the clinical effectiveness of DISCOVER on anxiety
4. To assess the clinical effectiveness of DISCOVER on wellbeing
5. To assess the effect of DISCOVER on sleep
6. To assess the effect of DISCOVER on resilience
7. To descriptively assess the accessibility of the workshops for hard-to-reach populations (e.g. ethnic minority students, those who have not previously accessed NHS services or school counselling)
8. To assess the acceptability of the intervention when workshops are run by CWPs or EMHPs
9. To examine how contextual factors (e.g. school environment) may have shaped the implementation of the experimental intervention, and how the intervention process (e.g. the conduct of workshop and follow-up) influenced the acceptability of the intervention to participants and contributed to the observed outcomes

### Trial design {8}

A two-arm single-blinded (researchers, analyst), UK-wide multi-centre cluster randomised controlled trial (cRCT) with 3- and 6-month follow-up. The trial will be a clinical effectiveness and cost-effectiveness evaluation and will take place in 60 secondary schools across the UK. The two parallel arms will be (i) a psychological stress workshop programme (DISCOVER), consisting of a 1-day CBT workshop, pre-workshop goal planning session, and 1–3 follow-up phone calls, and (ii) a control condition, chosen as a comparator to represent normal school provision. The unit of randomisation will be the school, thereby minimising contamination between intervention and control arms. Outcomes will be measured at baseline, 3-month follow-up, and 6-month follow-up in both study arms. Timings will fit around the school year, within a 3-year timeframe, to enable recruitment over 2 school years.

## Methods: participants, interventions, and outcomes

### Study setting {9}

The study will run in 60 secondary schools and sixth form colleges across England. The sites will be within London, Midlands, Northwest, and Southwest England.

Specific areas within each site are:

- London: Bexley, Enfield, Greenwich, Hackney, Islington, Newham
- Midlands: Burton, Shrewsbury, Solihull
- Northwest: Cheshire, Liverpool, Manchester
- Southwest: Bath and Northeast Somerset (BaNES), Bristol, Wiltshire

Clinical services will be initially approached by the 4 clinical site leads. Services will be working with some schools as part of the Mental Health in Schools Team (MHST) programme. Services will be recruited based on their staffing profile (one band 7 and two band 5 clinical staff) and willingness to participate in the trial. Schools will then be recruited based on their interest in the study as well as their willingness and ability to plan and organise the implementation of the trial together with the research team. Schools will be approached by site research staff. Recruitment will be limited to mainstream state schools and sixth form colleges with a minimum of 70 registered sixth form students.

### Eligibility criteria {10}

Cluster inclusion criteria for the trial are (i) secondary school with sixth form or dedicated sixth form college, (ii) state-funded, and (iii) sufficient resources (e.g. physical space) available to host the trial.

Cluster exclusion criteria are (i) further education college, (ii) privately funded school/college, and (iii) sixth form student population < 70.

Participant inclusion criteria for the trial are (i) aged between 16 and 18 years, (ii) attending school or college, (iii) sufficient English to provide valid informed consent and complete assessments in the BESST study, (iv) seeking psychological help for stress, (v) able to attend the DISCOVER workshop on school premises, and (vi) able to provide informed written consent to participate.

Participant exclusion criteria are (i) identified as actively suicidal through risk assessment, (ii) current involvement in psychological therapy for anxiety or depression with CAMHS, or (iii) severe learning difficulties.

### Who will take informed consent? {26a}

All potential participants will self-refer and be provided with an information pack, including the consent form when they attend a voluntary trial information meeting. They will be given at least 48 h to consider this information. If they decide to proceed, an individual meeting will be arranged with the site research worker to obtain informed written consent to participate.

### Additional consent provisions for collection and use of participant data and biological specimens {26b}

Additional consent provisions are not applicable for this trial.

## Interventions

### Explanation for the choice of comparators {6b}

Participating students in schools/colleges allocated to the control arm will receive their normal school care as well as ‘signposting’ information that provides them with a list of relevant resources available to them should they wish to seek further help. This control group was deemed most appropriate as it accurately represents the resources that would usually be available to participants outside of the trial intervention.

### Intervention description {11a}

#### Intervention arm

Students in the intervention arm will participate in the DISCOVER workshop shortly after randomisation.

##### Delivery method

DISCOVER is a brief, intensive, group workshop-based stress management programme for 16–18-year-olds, to which they can self-refer. The programme was developed in collaboration with a Teenage Advisory Group (TAG) of 16–18-year-olds with the aim of improving engagement, offering effective treatment, and maintaining participants’ motivation and improvement to reduce relapse. The collaborative approach also allowed the workshop to be developed to be acceptable across ethnic groups and both sexes. The workshop is a day-long face-to-face workshop, accommodating up to 19 students and taking place at school/college over a single day. Permission for students to attend and miss curricular activities is obtained from staff in advance. Each workshop is co-facilitated by one master’s/postgraduate diploma level therapist and 2 assistants with a college-level qualification, not necessarily in psychology (e.g. EMHPS, CWP), and delivered in accordance with the DISCOVER manual. The workshop programme includes CBT coping techniques for managing mood, anxiety, and stress, delivered in non-stigmatising language. Prior to attending the workshop, students meet individually with one of the three workshop leaders to think about their personal goals, which they set at the end of the workshop day. The workshop is then followed by up to 3 ‘goal-review’ telephone calls.

##### Core content

Each workshop begins with introductions and icebreakers. Psychoeducational content first focuses on a basic cognitive-behavioural model of emotional problems. A variety of presentation methods are used. Video clips involving teenage actors and group discussion are used to normalise young people’s experiences. Particular attention is given to personal, relationship, and academic stresses typical for the age group. CBT techniques for managing anxiety and mood problems are introduced and practised, supported by scripted role-plays, video demonstrations, and printed handouts. Behavioural strategies used include problem-solving and time management. Cognitive strategies include identification and challenging of negative thoughts.

##### Personalised follow-up

Participants are encouraged to set clear personal goals at the end of the workshop. After 1 week, participants are followed up individually by one of the workshop leaders in a 20–30-min telephone call. The purpose of this ‘telephone goal review’ is to monitor progress and support incorporation of CBT skills into real-life situations. If needed, participants are given the option of receiving 2 further telephone goal reviews within the 12-week post-workshop period. Participants will be offered a total of 1–3 telephone consultations in order to refine their original goal(s) and/or address unforeseen barriers. These will all occur before the participant meets with the blinded research worker for administration of the follow-up outcome measures.

#### Control arm

Participants within schools allocated to the control arm will not receive the DISCOVER workshop and will act as an inactive control, with access to normal school provision. At the start of the trial period, all participants will be provided with a signposting sheet detailing relevant resources and services available in the local area and online.

### Criteria for discontinuing or modifying allocated interventions {11b}

Research workers will monitor for potential harm during data collection sessions: A standard risk assessment and management protocol will be carried out by a research worker at baseline using the Ask Suicide-Screening Questions (ASQ) [41]. Additionally, in the intervention arm, workshop leaders will monitor for potential harm during workshop programme delivery and telephone contacts.

Any spontaneous or ASQ-related indications of risk will be referred to the trial manager, DISCOVER service lead, and school safeguarding lead, as appropriate. If the risk is judged to be ‘acute’ (i.e. in need of immediate safeguarding actions, as per usual clinical and school procedures), then the young person in question will be excluded from further study procedures and referred to the safeguarding team within the school.

Participants have the right to withdraw from the study at any time for any reason. The chief investigator (CI) also has the right to withdraw participants from the study in the event of SAEs or other reasons. As per the ‘Adverse event reporting and harms {22}’ section, all AEs/SAEs will be summarised and reported in the open report of the Data Monitoring Committee (DMC) which will also be circulated to the Trial Steering Committee (TSC). It is understood by all concerned that an excessive rate of withdrawals can render the study uninterpretable; therefore, unnecessary withdrawal of participants should be avoided. Should a participant decide to withdraw from the entire study, all efforts will be made to report the reason for withdrawal as thoroughly as possible. Should a participant drop out from attending the workshop programme, every effort will be made to continue to obtain follow-up data, with the permission of the participant. Participants who wish to drop out of the intervention will be asked to confirm whether they are still willing to provide data at any remaining follow-up assessments (and will be encouraged to do so).

### Strategies to improve adherence to interventions {11c}

To ensure adherence to intervention protocols, the research worker (RW) will meet with students individually to obtain informed consent and collect baseline data. The RW will also conduct the follow-up assessments. As the intervention workshop and assessment measures will be carried out with a member of the trial team present, this will allow us to ensure all protocols are fully adhered to.

To ensure adherence to follow-up measures, each student will be contacted by the trial manager, and via designated school staff, (up to 5 times) for the 3- and 6-month follow-up assessments, with visit windows of ± 4 weeks for the 3-month follow-up assessments and ± 6 weeks for the 6-month follow-up assessments. However, the research team will endeavour to ensure data is collected as closely as possible to the 3- and 6-month time points. Should follow-up appointment reminders fail, non-responders will be sent assessment questionnaires along with instructions for completion and asked to return these by post. In order to protect blinding, the RWs will remind the students at the start of each assessment that they are not to divulge whether they received the workshop or not.

In an effort to reduce attrition bias, and maintain good adherence to the study protocol, an incentive of Amazon vouchers will be offered to students who consent to take part in the study (£15) as well as complete measures at other time points (£15 for 3 months and £25 for 6 months).

### Concomitant interventions {11d}

Other treatments including medication are permitted in both arms of the trial. This will be recorded using the Child and Adolescent Service Use Schedule (CA-SUS), described below, completed by the participant with guidance from the RW.

### Provisions for post-trial care {30}

There is no explicit post-trial care outside of the signposting information provided to all participants and any further care provided by the school safeguarding team if risk is identified during the trial.

### Outcomes {12}

#### Primary endpoint

The primary endpoint will be the depression symptoms in the intervention arm, post-intervention at 6 months post-randomisation, compared to the control arm. This will be assessed using the long version of the Mood and Feelings Questionnaire (MFQ) [42]. The MFQ is a 33-item self-report depression measure, which has displayed good validity and reliability amongst adolescent samples. Scores range from 0 to 66, with a clinical cut-off of > 27.

#### Secondary endpoints

1. Feasibility of the intervention based on satisfactory (green light) outcome of an internal pilot.
2. Anxiety symptoms in the intervention arm, post-intervention at 6 months, compared to the control groups. This will be assessed using the Anxiety Sub-scale from the Revised Child Anxiety and Depression Scale (RCADS) — child version [43]. This is a 47-item self-report measure. It has good construct validity, internal consistency, and test re-test reliability.
3. Wellbeing scores in the intervention arm, post-intervention at 6 months, compared to the control groups. This will be assessed using the Warwick Edinburgh Mental Wellbeing scale (WEMWBS) [44], a 14-item self-report measure of mental well-being, successfully used with adolescents [45].
4. Sleep quality in the intervention arm, post-intervention at 6 months, compared to the control groups. This will be assessed using the Sleep Condition Indicator (SCI) [46]. The SCI is a brief 8-item scale which measures sleep problems against the DSM-5 criteria for insomnia disorder. The SCI is valid, reliable, and sensitive to change.
5. Resilience in the intervention arm, post-intervention at 6 months, compared to the control groups. This will be assessed using the Child and Youth Resilience Measure 12 (CYRM-12) [47], which is a 12-item scale designed as a screening tool to explore the resources (individual, relational, communal, and cultural) available to individuals, that may bolster their resilience.
6. Accessibility of the intervention for hard-to-reach populations (ethnic minority students) based on demographic information collected at baseline. The ethnicity data of students consented to the study will be compared with local norms at the regional level.
7. We will measure student satisfaction using the Client Satisfaction Questionnaire (CSQ-8) [48] and conduct additional assessments of the acceptability of the intervention when workshops are run by CWPs using student feedback forms.
8. To examine how contextual factors (e.g. school environment) may have shaped the implementation of the experimental intervention, and how the intervention process (e.g. the conduct of workshop and follow-up) influenced the acceptability of the intervention to participants and contributed to the observed outcomes.

#### Economic evaluation parameters

1. Health-related quality of life assessed with the EQ-5D-3L [49], used to calculate quality-adjusted life years (QALYs) for use in economic evaluation. The 3-level version of the EQ-5D was selected, rather than the expanded 5-level version (EQ-5D-5L), because it was the version tested in the feasibility study and found to be acceptable and because there is evidence of the validity of the EQ-5D-3L for use with adolescent depression populations [50], which is not available for the EQ-5D-5L.
2. Use of health and social care services measured using the Child and Adolescent Service Use Schedule (CA-SUS), designed for, and successfully implemented in, multiple evaluations of interventions for children and young people with mental health conditions, including depression [51, 52]. The CA-SUS collects information on the use of all hospital and community-based health and social care services, including those provided in education settings, prescribed medications, and Local Authority provided accommodation.

#### Process evaluation parameters

The process evaluation parameters will be as follows:

1. Student feedback on workshops: A feedback form will be completed at the end of the workshop, with open-ended questions about the workshop that were (i) liked, (ii) disliked, and (iii) most helpful and (iv) could be improved.
2. Techniques learned during the workshops and used by students in a 3-month period following the interventions, which will be recorded during follow-up phone calls.
3. Semi-structured qualitative interviews will be conducted by the research workers in 8 intervention schools using semi-structured interviews with students (*n*=16) and workshop facilitators (*n*=8). We will also conduct focus groups with school staff (*n*=8). Students will be selected purposively within each of the 8 sampled intervention arm schools to represent the range in terms of self-reported engagement with the workshop (as reported through the student feedback questionnaire). We will also seek to be inclusive in relation to gender and ethnicity. Data collection will be scheduled for the period post-intervention, post-6-month follow-up, and post-exams. This means that students will be able to provide a reflective account of their participation in the workshop.

### Participant timeline {13}

**Fig. 1:**
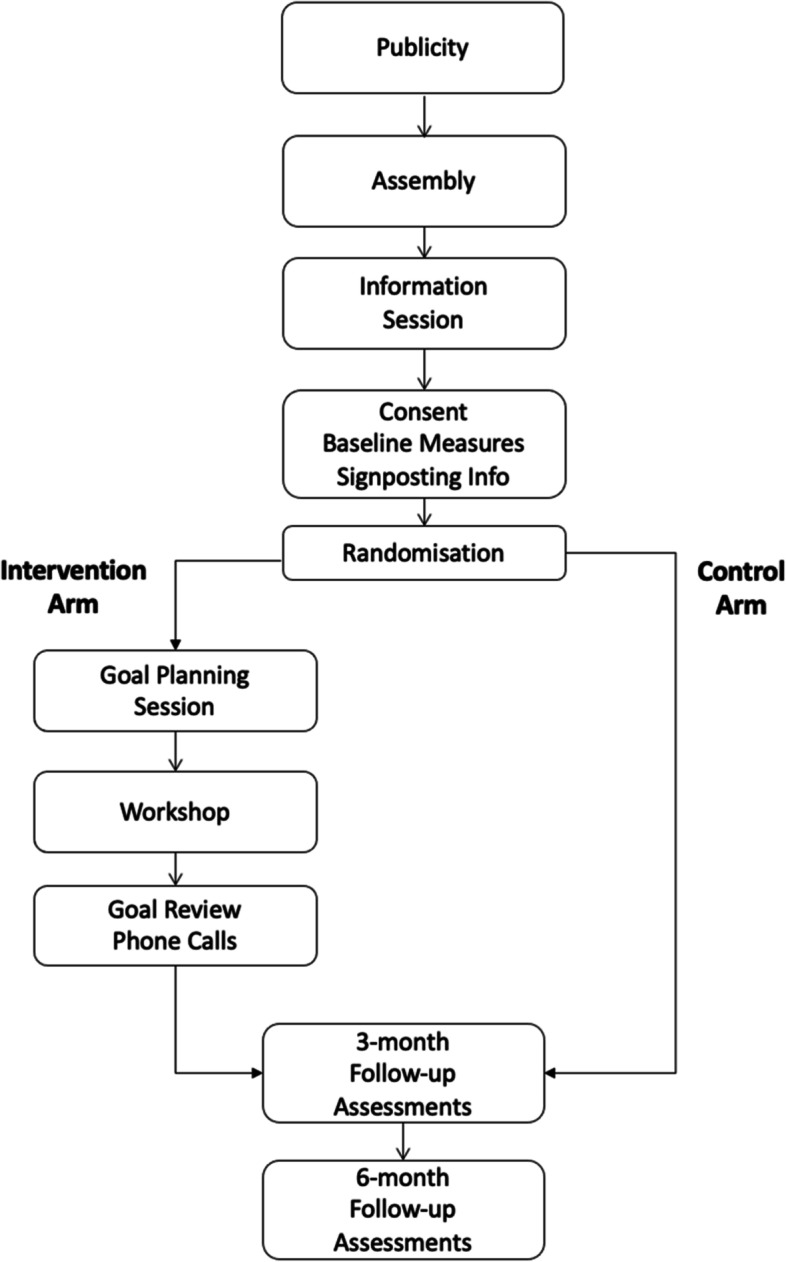
Participant flow through the trial. Note: This flowchart depicts the trial timeline within each school and college

The trial will be publicised within schools and colleges from the start of the academic year. Potential participants will be invited to provide informed consent prior to randomisation of schools. Baseline measures will be collected in the 2 weeks prior to randomisation. Workshops will take place in the month following randomisation. The first follow-up measures will be obtained approximately 3 months after randomisation in both arms of the trial. The second follow-up measures will be collected approximately 3 months after the first follow-up (Fig. 1 and Table 1).

**Table 1:** Participant measures completed at each stage of the trial

	Publicity at school	Study presentation	RW meets students and pre-measures	Pre-workshop goal planning meetings	Intervention	Post-intervention measures	Goal review follow-up calls	3-month follow-up measures	6-month follow-up measures	Interviews
6th form assembly	X									
Lunchtime information meeting		X								
Consent			X							
Socio-demographics			X							
Ask Suicide-screening Questions (ASQ)			X							
Mood and Feelings Questionnaire (MFQ)			X					X	X	
Revised Child Anxiety and Depression Scale			X					X	X	
Warwick Edinburgh Mental Wellbeing Scale			X					X	X	
Sleep Condition Indicator			X					X	X	
Child and Youth Resilience Measure			X					X	X	
Student feedback form						X (Exp arm only)				
Client Satisfaction Questionnaire						X (Exp arm only)				
Techniques used							X (Exp arm only)			
Child & Adolescent Service Use Schedule (CA-SUS)			X					X	X	
EQ-5D-3L			X					X	X	
Intervention					X (for schools in Exp arm only)					
Goal planning				X (Exp arm only)	X (Exp arm only)		X (Exp arm only)			
Process evaluation										X (Exp arm only)

### Sample size {14}

The sample size was calculated based on the results of the feasibility study. Based on these results [38], where estimated intraclass correlations (ICCs) were found to be negligible (between 0 and 0.003), we estimate that to detect a mean change score of 5.6 with alpha 0.05 on the Mood and Feelings Questionnaire [42] in the intervention group and 2.8 in the control group with SD = 10 (effect size 0.28), we need an ICC of 0.02. However, we have increased the ICC from 0.02 to 0.03 which is consistent with typical ICC found from other studies of mental health interventions of mood outcomes found in schools in the UK [53]. This has increased the number of schools from 54 to 60, increasing the number of students from 810 to 900, with 15 students per school (average). This will give 90% power to detect differences. This assumes a loss to follow-up of 12.5% of students, and 4% (*N* = 2) schools dropping out, based on the low dropout rate found in the feasibility study.

### Recruitment {15}

#### School recruitment

Sixty schools/sixth form colleges will be recruited into the trial. Approximately half of these sixth forms will be recruited to participate in the first year of data collection (cohort 1), with the other half of schools recruited to participate in the second year of data collection (cohort 2).

#### Participant recruitment

Targeted communications, augmented by posters and flyers, will be used to publicise the study at 60 schools and sixth form colleges. Students will then be informed at a school assembly about the study’s aims and methods, where a research worker will give a brief presentation about the study. Where possible, a male role model from within the school staff will also be present, for the purpose of promoting male engagement. Students will be invited to register their initial interest by attending a further small group information meeting. The information meeting will be offered during a lunchtime session at each school. Students will be reminded in advance by the relevant teachers. The session will be run by a research worker, and where possible, teachers and male role models will also be in attendance. Students will be shown a presentation giving more detailed information about the BESST trial and what they would need to do if they decided to take part in the trial. They will also be given a written Participant Information Sheet. It will be made clear that schools will be randomised into experimental and control schools following the first session of outcome measures and only 50% schools would receive the DISCOVER workshop programme. It will be explained to students that they receive a total of £55 gift vouchers for participating, regardless of which arm their school is randomised to. It will also be made clear that if more than the maximum number of students come forward, students will be randomly allocated to participate in the trial; those who are not allocated to participate will be provided with a signposting information document. There will also be opportunities for students to ask questions. Students who are unable to attend the group information meeting will be offered further information and the opportunity to ask questions either through their teacher or a research worker. Students will have at least 48 h after the information meeting to decide whether or not they wish to proceed. If they decide to proceed, an individual meeting will be arranged with the research worker to obtain informed written consent to participate and complete the assessment measures. Parents will only be informed about this decision when specifically requested by the young person. Student participation will be discontinued if they decide to withdraw at any point.

#### Patient and public involvement (PPI)

Adolescent PPI groups from the Anna Freud Centre in London have been consulted with to inform effective recruitment strategies. PPI members have advised on the content and delivery of participant recruitment presentations to provide optimal clarity of trial information and maximise engagement of the presentations. These approaches will be implemented when delivering participant recruitment sessions.

## Assignment of interventions: allocation

### Sequence generation {16a}

To reduce selection and recruitment bias, schools will be randomly allocated to experimental or control arms in a 1:1 ratio after participants have provided informed consent and completed baseline measures. The sequence will be generated by the Kings Clinical Trials Unit (KCTU)-affiliated statisticians using block covariate minimisation for deprivation and school size stratified by site, developed by Carter and Hood [54]. Cluster allocation will then be communicated to the trial manager, who will then inform schools.

### Concealment mechanism {16b}

As randomisation is performed after participant baseline data is collected by site, the allocation sequence will be concealed until clusters are assigned allocations and these allocations are communicated to the trial manager. The sequence will be generated following a randomisation protocol that will ensure that the senior statistician remains blinded throughout the duration of the study.

### Implementation {16c}

Participants will be enrolled and consented by the research workers. If more than 19 students in any school are consented, a random number generator will be used to randomly select students to take part in the study, due to constraints in the number of workshops that could be run. Students not continuing into the trial will be provided with a signposting information sheet.

Prior to randomisation of a cluster, participant consent and baseline data will be collected. Aggregate baseline covariate data of the clusters within a site will be gathered by the research workers for collation by the trial manager. Once cluster-level covariate data within a site and participant-level baseline data has been collected, randomisation can be performed. The trial manager will communicate the covariate data to the KCTU-affiliated statisticians who will then generate the sequence. This allocation list will then be communicated to the trial manager who holds the randomisation key assigning A or B to intervention in order to implement the assigned allocations within the clusters. This process will continue per site until all clusters have been allocated for cohort 1.

The cohort 2 school allocation sequence will be generated using the same procedure in the knowledge of the cohort 1 allocation to provide balance across both cohorts. Once the sequence is generated, a PDF of the allocations will be stored as source data, and the A/B allocations will be entered into MACRO.

## Assignment of interventions: blinding

### Who will be blinded {17a}

Research workers who are directly involved in data collection will remain blind to cluster allocation. Several steps will be taken to preserve blinding. First, blinded research workers will have minimal contact with workshop leaders prior to follow-up data collection. Second, unblinded members of the research team will liaise with research sites and participants to confirm practical arrangements for data collection, thereby minimising contact between blinded researchers and schools following baseline measures. Third, blinded research workers will use a standardised script during data collection to remind students not to disclose their allocation status. The blinded research workers will also display signs, containing similar reminders, during follow-up visits to schools. The trial manager will be unblinded, as they will coordinate the delivery of the DISCOVER programme. After approval of the first draft of the statistical analysis plan (SAP) and health economic analysis plan (HEAP), the trial statistician and trial health economist will become pseudo blinded to the allocation coded as A or B. The trial health economist will become unblinded upon database lock to allow the intervention to be costed for those in the intervention group. The chief investigator, senior trial statistician, and senior health economist will remain blinded until the database lock. At which point, they will be pseudo blinded (aware of arms as A and B) until the analysis is fully interpreted. Any incidents of unplanned unblinding will be recorded.

### Procedure for unblinding if needed {17b}

Participants will be aware of the allocated arm that they receive. Researchers completing follow-up will be blinded to the allocation. It is unlikely any AE will be linked to the intervention and therefore participants will not be asked explicitly about the allocation arm. However, if further information is required to determine the relatedness of the event to the allocation, the trial manager, who will not be blinded to allocation, will follow up directly with the participant.

## Data collection and management

### Plans for assessment and collection of outcomes {18a}

#### Primary and secondary outcomes

These measures will be recorded at pre-intervention baseline and 3- and 6-month post-randomisation follow-up time points. Research workers will be trained to facilitate these measures using questionnaire booklets. At each assessment time point, the local research worker will attend a pre-arranged 1–2-1 appointment with each research participant. The appointment will take place in a private room at the participants’ school. During the appointment, the research worker will explain each measure to the participant and then allow the participant to complete the measure whilst remaining present for any questions. The participant will be briefed by school staff prior to each appointment not to reveal the workshop allocation of the school in order to keep the research worker blinded. The research worker will also remind the participant of this at the start of each appointment.

Should follow-up appointment reminders fail, non-responders will be sent assessment questionnaires along with instructions for completion and asked to return these by post. For participants that are difficult to schedule in-person due to school term dates, we will provide them with assessment questionnaires along with a stamped addressed envelope to return the questionnaires to the study team.

#### Process evaluation parameters

The student feedback form will be provided to the participants by the workshop delivery team members at the end of each workshop and will be completed at that time. The CSQ-8 [48] described amongst the trial outcomes will also be administered at this point.

Techniques used in a 3-month period following the interventions, which will be recorded during follow-up phone calls. Phone calls will be conducted by the workshop delivery team as part of the overall workshop programme. These phone calls are scheduled during the period following the workshop, prior to the 3-month follow-up assessments. During these phone calls, the workshop delivery team member will ask the participant which workshop techniques they have been using during this period and manually record their responses against a list of all workshop techniques.

The qualitative process evaluation will be conducted by the research workers in 8 intervention schools using semi-structured interviews (with *n* = 16 students, *n* = 8 workshop facilitators) and focus groups with school staff (*n* = 8). These will be conducted in the June–August period after all other follow-up measures have been completed.

### Plans to promote participant retention and complete follow-up {18b}

Before the 3-month follow-up, participants will receive a series of reminders from school staff about the timing of follow-up assessments. Participants (intervention arm only) will receive a text message following the workshop to acknowledge their attendance and arrange the next steps (i.e. telephone goal reviews). Further reminders will be provided during telephone goal reviews. Emails will be sent to the relevant school staff in the weeks prior to follow-ups in order to schedule appointments and prompt them to remind students.

Participants will receive vouchers at three time points throughout the study as a thank you for their continued involvement. A total of £55 will be given to each participant. The first £15 will be provided following the completion of baseline measures, another £15 will be provided at the 3-month follow-up, and the final £25 will be provided at the 6-month follow-up.

### Data management {19}

All structured personal data in the form of physical, identifiable files (e.g. completed self-report questionnaires, demographic measures, and consent forms) will be stored in a locked cabinet drawer within a secure research office at King’s College London. Research data will be entered into a secure electronic data capture (EDC) MACRO database developed following King’s Clinical Trials Unit (KCTU) database development, test, and validation standard operating processes.

Data will be centrally checked for both completeness and errors and pattern missingness assessed. Any potential errors are sourced by back to sites to confirm the correct data. The data held on the database will be compared to 20% of the source data by a research worker from a different site to ensure accuracy. Data is checked and signed off by each site lead prior to database lock.

KCTU Statistics and health economic SOPs will be followed on data manipulation, analysis, and quality assurance. All procedures will be in line with GCP and GDPR.

### Confidentiality {27}

Any identifiable information such as names and contact details will be removed from completed measures. An anonymous code will be assigned to each participant to identify the completed measures. Pseudonyms will be used in interview transcripts where participants mention names, places, or any other identifiable information. All data will be anonymised before any reporting takes place. Only staff with direct teaching/pastoral responsibility will be informed about students’ participation in the trial in order to protect confidentiality. School staff will not have access to the DISCOVER workshop or students’ responses to measures except in the case of assessed suicidal intention using the ASQ [41].

### Plans for collection, laboratory evaluation, and storage of biological specimens for genetic or molecular analysis in this trial/future use {33}

Not applicable, no samples were collected.

### Analysis {20a}

A statistical analysis plan will be generated following the King’s Clinical Trials Unit (KCTU) standard operating procedures for Statistics (KCTU Statistics) and will contain a detailed description of the planned analysis. The first draft will be approved whilst both the trial statistician and senior statistician are blinded to the allocation of schools.

#### Primary outcome analysis

MFQ scores will be analysed using a multilevel linear model adjusted by the following fixed effects: (1) aggregated level school deprivation, (2) geographical area, (3) school size, (4) gender, (5) ethnic group, (6) dummy variable indicating treatment group, and (7) baseline measure of the outcome (MFQ score). A treatment group by time interaction term will also be included to allow for extracting comparisons at both follow-up times. A random intercept model will be fitted for each school and student, and the difference between the intervention and control MFQ score will be estimated, alongside the 95% confidence interval and *p*-value. The primary endpoint for MFQ analysis is a 6-month follow-up.

#### Secondary outcome analysis

A multilevel adjusted multivariable regression will be used for continuous outcomes, and a multivariable logistic regression for binary outcomes will be used across the 3- and 6-month time points.

The following outcomes will be analysed as binary outcomes, using a multilevel logistic regression: (1) MFQ (clinical cut-off at > 27), (2) adverse events, and (3) serious adverse events.

The following outcomes will be analysed as continuous outcomes using a multivariable regression: (1) EQ-5D-3L, (2) RCADS, (3) WEMWBS, (4) SCI, and (5) CYRM-12.

The above analyses will be adjusted for covariates consistent with the primary outcome.

#### Health economic analyses

A health economic analysis plan will be generated following the King’s Clinical Trials Unit (KCTU) standard operating procedures for Health Economics (KHE-01 Health Economics Standard Operating Procedure V2.0) and will contain a detailed description of the planned analysis. The first draft will be approved whilst both the trial economist and senior economist are blinded to the allocation of schools.

The primary economic analysis will be a cost-utility analysis at the 6-month follow-up with effectiveness measured in terms of QALYs using the EQ-5D-3L and taking the NHS/personal social services perspective preferred by NICE. A secondary economic analysis will explore cost-effectiveness using the primary clinical outcome measure, the MFQ.

Data on the DISCOVER programme will be taken from clinical records. The cost of the DISCOVER programme will be calculated using a detailed, micro-costing approach [55]. The salary costs of the group facilitators including employer on costs (national insurance and superannuation) and appropriate overheads (capital, management, administration, etc.) will be weighted to include relevant non-face-to-face time spent on other activities (e.g. session preparation, report writing, meetings, training, etc.). The cost of the DISCOVER programme will be allocated across all young people invited to attend on the basis that the workshops are closed groups and will go ahead irrespective of attendance [56]. All other health and social care services, measured using the CA-SUS, will be costed using nationally applicable unit costs (e.g. PSSRU Unit Costs of Health and Social Care compendium, NHS Reference Costs for hospital contacts, British National Formulary for medications).

Quality-adjusted life years (QALYs) will be calculated by multiplying EQ-5D-3L weights [57] by the time between baseline and the 3- and 6-month follow-up points, assuming linear change between periods and using the area under the curve approach [58].

Costs and outcomes will be compared in terms of mean differences and 95% confidence intervals from non-parametric bootstrap regressions (1000 replications) to account for non-normal distribution common to economic data. Cost-effectiveness will be explored in terms of cost per QALY using incremental cost-effectiveness ratios [59] with uncertainty represented by cost-effectiveness acceptability curves [60]. All analyses will be adjusted in line with the clinical analyses by (1) aggregated level school deprivation, (2) geographical area, (3) school size, (4) gender, (5) ethnic group, and (6) baseline severity (MFQ score, plus the variable of interest, e.g. baseline cost and/or utility). Missing data will be dealt with in line with the clinical analyses (i.e. using the approach recommended by Jakobsen [61]), with further detail described in the HEAP. If appropriate, sensitivity analyses will explore the impact of (1) missing data (e.g. a complete case analysis) and (2) influential outliers [62].

The primary analysis will be on the intention-to-treat (ITT) population using a linear mixed model which allows for missing data under the missing at-random assumption using the approach recommended by Jakobsen [61], and further detail will be described in the SAP. If there is a positive difference in effectiveness between intervention and control at a 6-month follow-up, we will develop a decision analytical Markov model to extrapolate study costs and QALYs over a longer period, using available data from the literature and appropriate longitudinal databases.

### Interim analyses {21b}

After completion of year 1, we will assess the feasibility of continuing the BESST trial, presenting the findings of an internal pilot to the DMC. We will compare our recruitment, retention, and fidelity, and only progress to the full trial by meeting the following ‘Go’/’No go’ criteria, as indicated by meeting the green criteria. Less than this value will result in being flagged as amber and or red, as per the boundaries listed in Table 2.

**Table 2:** Feasibility parameters for the BESST trial internal pilot

	Red*	Amber	Green
Randomising 19 schools	< 15	15 to 18	≥ 19
3- and 6-month follow-up measures conducted in 80% of randomised schools	< 12	12 to 15	≥ 15
Recruit 180 students to the trial	< 144	144 to 179	≥ 180
Participant adherence (% of students followed up)	< 64%	64 to 79%	≥ 80%
60% of students from the intervention arm will give satisfaction ratings on the CSQ-8 of at least 26 points	< 48%	48 to 59%	≥ 60%

The trial may be prematurely discontinued by the sponsor or chief investigator on the basis of new safety information or for other reasons given by the Data Monitoring & Ethics Committee/Trial Steering Committee regulatory authority or ethics committee concerned. The trial may also be prematurely discontinued due to a lack of recruitment or upon advice from a Trial Steering Committee (if applicable), who will advise on whether to continue or discontinue the study and make a recommendation to the sponsor. Furthermore, if results from the internal pilot demonstrate the trial is not meeting the aims outlined, the results will be presented to the DMC, and they will advise on whether the trial should continue or be stopped. If the study is prematurely discontinued for any reason, active participants will be informed, and no further participant data will be collected.

### Methods for additional analyses (e.g. subgroup analyses) {20b}

#### Qualitative analysis

The qualitative interviews conducted as part of the process evaluation are designed to (a) examine the contextual and process factors that either support or obstruct the implementation of the intervention, (b) examine the experience of participants and workshop facilitators, and (c) assess whether and how the contextual (e.g. school environment) and process factors (e.g. publicity) identified through this work influence the intermediary outcomes (e.g. engagement, intervention fidelity, adherence to intervention protocol) as well as the primary and secondary outcomes assessed in the trial. This qualitative work will be conducted in 8 intervention schools using semi-structured interviews (with *n* = 16 students, *n* = 8 workshop facilitators) and focus groups with school staff (*n* = 8).

Semi-structured interviews and focus groups will be conducted in a purposive sample (*n* = 8) of intervention arm schools sampled to represent range and diversity in terms of school type, geography, socio-economic profile, and record of recruitment and attrition at 3 months.

##### Qualitative sub-study 1 {20b}

Two students will be purposively selected from each of the 8 intervention arm schools (*n* = 16) to represent a range in terms of gender and ethnicity. Interviews will be conducted as soon as is practical after the 6 month trial follow-up assessment (and student examinations). Hence, participants will provide a retrospective account and will be invited to provide a reflective account of their experience of the DISCOVER workshop and whether and how they perceive/experience any benefit.

##### Qualitative sub-study 2

Semi-structured interviews will take place with eight workshop facilitators. These will investigate their experience of delivering the workshop and whether contextual factors or aspects of the process are perceived to contribute to intermediary outcomes and the primary and secondary trial outcomes.

##### Qualitative sub-study 3

We will conduct staff focus groups with up to six participants in 8 schools to explore local implementation, the experience of teacher training, and perceptions of the conduct and impact of groups.

### Methods in analysis to handle protocol non-adherence and any statistical methods to handle missing data {20c}

A protocol deviator (PD) is defined as a breach from the protocol that is unlikely to influence the findings of the study. Where a PD is carried out, it will be noted with the Trial Management Group (TMG) minutes. A protocol violator (PV) is a breach from the protocol that may result in a change to the study findings. An incident that may result in a PV would be a participant not adequately fulfilling adherence to the workshop or completing the primary outcome assessment outside of the study window.

Missing data will be explored. The primary analysis will be on the intention-to-treat (ITT) population using a linear mixed model which allows for missing data under the missing at-random assumption using the approach recommended by Jakobsen [61]; further detail will be described in the SAP.

### Plans to give access to the full protocol, participant-level data, and statistical code {31c}

The chief investigator will act as custodian of the data in accordance with legislation and the terms of the research sponsor (King’s College London) and funder (National Institute for Health Research, UK).

Trial-related monitoring, audits, Research Ethics Committee (REC) review, and regulatory inspections will be permitted by providing the sponsors and REC direct access to source data and other documents providing this does not infringe upon data protection obligations and participants’ right to confidentiality. The datasets generated and analysed and the corresponding statistical code will be available in anonymised form from the research team on reasonable request, subject to review, following the publication of trial results.

## Oversight and monitoring

### Composition of the coordinating centre and trial steering committee {5d}

Regular Trial Management Group meetings will be organised throughout the course of the trial. The TMG will be chaired by the CI (or delegate), and members will compromise as each co-applicant, the trial manager, junior statistician, trial administrator, and site research workers. The trial conduct will be discussed and organised at the TMG.

An independent Trial Steering Committee (TSC) will be established to monitor progress, advise the investigators in general scientific and management issues, and ensure that there are no major deviations from the study protocol. The TSC will include an independent chair, an independent statistician, and at least 5 other independent members with research and clinical experience with young people with mental health problems and/or school mental health. The TSC will also include two Young Advisors from the PPI group. The TSC will meet at least once per year. The lead applicant will inform the TSC Chair who may call additional meetings when there are matters arising from the conduct or management of the trial that might require their advice.

### Composition of the data monitoring committee, its role, and reporting structure {21a}

The Data Monitoring Committee (DMC) will review the on-going safety profile of the intervention and be the only committee able to identify the on-going data. The DMC will consist of a clinical chair and independent statistician, and one additional independent member, with the CI as an observer. An open DMC report will be prepared by the trial statistician. The DMC will make a recommendation to the TSC prior to a TSC meeting about the continuation of the trial. The DMC will review recruitment, retention, data quality, the primary clinical result, and adverse events. The DMC will meet at least annually.

### Adverse event reporting and harms {22}

BESST is a low-risk non-medical trial. Research workers from each site will note adverse events (AEs)/serious adverse events (SAEs) at each follow-up interview and enter these into the MACRO database, and any SAEs or suspected SAEs that are recorded will be reported to the trial manager. Facilitators will record AEs/SAEs from the workshop and follow-up calls; all these events will be reported to the trial manager to enter onto the trial database. All AEs/SAEs will be summarised and reported in the open report of the DMC which will also be circulated to the TSC. SAEs will also be circulated to the DMC chair for review. Action will then be taken accordingly depending on implications for the conduct of the trial.

We do not anticipate safety concerns arising as a direct result of the workshop programme, which is usually perceived as helpful by students. However, we will monitor adverse events carefully as one of our outcomes and ensure they are appropriately documented and addressed. Any that arise as a result of the workshop programme, however unlikely this may be, will be escalated to the independent DMC for review and opinion as to necessary adjustments to protocol. Adverse events of any kind will also be reported to the school safety officer, following school safety procedures.

Adverse events that are pre-existing and expected prior to the planned delivery of the DISCOVER programme (or matched delivery for participants in the usual care schools) will not be reported to the DMC.

### Frequency and plans for auditing trial conduct {23}

Monthly recruitment reports will be submitted to the funder. A pilot report after the completion of cohort 1 will be submitted to the funder to outline trial progress.

### Plans for communicating important protocol amendments to relevant parties (e.g. trial participants, ethical committees) {25}

All protocol amendments will be immediately communicated to relevant parties in writing. The amended protocol will be uploaded to the relevant trial registries.

### Dissemination plans {31a}

We will disseminate our academic findings through high-impact open-access publications. Our main trial findings will be of national and international interest, and we aim to publish them in high-impact, peer-reviewed, open-access journals (e.g. *Lancet*, *BMJ*). We will ensure that the trial outcome is known to clinicians and academics by presenting our findings at relevant national meetings (e.g. Association of Child and Adolescent Mental Health) and International Conferences (World Congress of Behavioural and Cognitive Therapy; European Association of Cognitive and Behavioural Therapy).

Results will be made available to all participants and presented to teachers after the completion of the study. Young Advisors, as part of DISCOVER PPI, will help draft the results in different styles and formats using different media (video-blogs, tweets) that will be accessible for young people and their carers. Wide service user and public audiences will access background information and reports through a designated project website, and the contents and features of which will be co-produced by Young Advisors. We will also raise public awareness of our results through press releases and the media.

We will share our results with policymakers through the Improving Access to Psychological Therapies Programme for Children and Young People (IAPT-CYP) which is a national training programme which oversees the development of CAMHS training in evidence-based interventions. We will also disseminate our findings to the Department for Education and Department of Health in relation to the new national Green Paper initiatives, which detail plans for national-level frameworks to train graduate mental health workers who specifically run interventions in schools. This is particularly relevant to the dissemination of DISCOVER given that we are testing the delivery with a mix of clinical psychologists and 2 CWPs/EMHPs. We will also disseminate our findings to schools and academies, relevant education conferences, teacher training, clinical psychology training courses, and CAMHS courses, e.g. MSc in Child Mental Health at KCL.

## Discussion

Previous research demonstrates a lack of easily accessible and cost-effective interventions for 16–18-year-olds seeking help for depression and anxiety. Long waiting times, stigma, inconvenient appointment times, transportation difficulties, and high thresholds for specialist referral are all barriers to young people receiving the care they need. The DISCOVER workshop programme addresses these barriers to provide a much-needed, easily accessible, resource that has been formulated specifically for this demographic.

As evidenced by our prior research investigating the feasibility of this programme in London schools, the intervention appeared accessible and acceptable to 16–18-year-olds. It also appeared to engage under-represented groups. The feasibility trial obtained results tentatively suggesting it also results in significant reductions in depression and anxiety.

We are now able to progress to the next stage of development and testing, with a full UK-wide clinical trial of the DISCOVER intervention with a much larger group of students in a fully powered cluster randomised controlled trial. The Brief Educational Workshops in Secondary Schools Trial (BESST) will allow us to extend our existing findings by examining 6-month outcomes of the intervention on depression and anxiety, examine mediators and moderators of change, and conduct a health economic evaluation to assess the cost-effectiveness of the intervention. This trial will also be able to generate much more robust evidence about accessibility, reach, acceptability, and impact. And a qualitative study is being carried out to examine processes leading to effectiveness.

Should these evaluations demonstrate positive findings, DISCOVER could provide a service model that can be utilised across the UK in school-based mental health provision to achieve a much-needed and very significant impact on the mental health of this adolescent age group.

## Trial status

Protocol version 1.2.2. Recruitment began on 1 September 2021 and will be completed by 31 December 2022.

## Supplementary Information

**Additional file 1.** Brief Educational workshops in Secondary Schools Trial (BESST).

## Data Availability

The chief investigator (JB) will act as custodian of the data in accordance with legislation and the terms of the research sponsor (King’s College London) and funder (National Institute for Health Research, UK). The investigator(s) will permit trial-related monitoring, audits, REC review, and regulatory inspections by providing the sponsor(s) and REC direct access to source data and other documents providing this is within the bounds of data protection and the protection of participants’ confidentiality. The primary analysis of the trial data will be performed by the trial statisticians and health econometricians. Other members of the team will have access to the data to perform analyses in accordance with a publication policy agreement.

## Abbreviations

BESST: Brief Educational workshops in Secondary Schools Trial
RCT: Randomised control trial
NIHR: National Institute for Health Research
HTA: Health Technology Assessment
CAMHS: Child and Adolescent Mental Health Services
CBT: Cognitive-behavioural therapy
QALY: Quality-adjusted life year
CWP: Children and young people’s Wellbeing Practitioner
EMHP: Educational Mental Health Practitioner
NHS: National Health Service
CRCT: Cluster randomised control trial
MHST: Mental Health School Team
RW: Research worker
CA-SUS: Child and Adolescent Service Use Schedule
MFQ: Mood and Feelings Questionnaire
RCADS: Revised Child Anxiety and Depression Scale
WEMWBS: Warwick Edinburgh Mental Wellbeing Scale
SCI: Sleep Condition Indicator
CYRM-12: Child and Youth Resilience Measure
CSQ-8: Client Satisfaction Questionnaire
ICC: Intraclass correlation
ASQ: Ask Suicide-screening Questions
EDC: Electronic data capture
KCTU: Kings Clinical Trials Unit
SAP: Statistical analysis plan
PSSRU: Personal Social Services Research Unit
PD: Protocol deviation
PV: Protocol violation
TMG: Trial Management Group
TSC: Trial Steering Committee
DMC: Data Monitoring Committee
PPI: Patient and public involvement
CI: Chief investigator
AE: Adverse event
SAE: Serious adverse event
REC: Research Ethics Committee
SOP: Standard operating procedure
