# Supplementary material for: Brief Educational Workshops in Secondary Schools Trial (BESST): protocol for a school-based cluster randomised controlled trial of open-access psychological workshop programme for 16–18-year-olds
Source: Trials. 2022 Nov 9;23:935. doi: 10.1186/s13063-022-06830-8 (PMC9647960; doi:10.1186/s13063-022-06830-8)
Supplement: Supplementary file 1 — Additional file 1. Brief Educational workshops in Secondary Schools Trial (BESST). [file 13063_2022_6830_MOESM1_ESM.pdf]

# Brief Educational workshops in Secondary Schools Trial (BESST)

Early intervention for depression and anxiety in 16–18-year-olds: a multi-centre, cluster randomised control trial of a self-referral psychological stress workshop programme in schools

## Statistical Analysis Plan

## Version V1.2

ISRCTN: 90912799. Protocol version: 1.2.3.

| Version | Completed / amended by | Role | Date |
|---|---|---|---|
| V1 | Kirsty James | Statistician (KCL) | 10.04.2019 |
| V1.1 | Kirsty James / Jodie Lord | Statistician (KCL) | 15.08.2022 |
| V1.2 | Jodie Lord | Statistician (KCL) | 19.08.2022 |

Trial statistician: Jodie Lord

Signed: Date: 15/08/2022

Trial statistician: Kirsty James

Signed: Date: 15/08/2022

Senior trial statistician: Ben Carter

Signed: Date: 15/08/2022

Chief Investigator: June Brown

Signed: 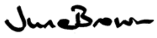 Date: 15aug22

TSC Statistician: Siobhan Creanor

Signed: 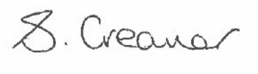 Date: 19/08/2022

*A) QUANTITATIVE ANALYSIS PLAN 5*

*1. Description of the trial 5*

**1.1 Principal research objectives to be addressed 5**
1.1.1 Primary objective 5
1.1.2 Secondary objectives 5

**1.2 Trial design including blinding 6**

*Figure 1. Trial design flow diagram 7*

**1.3 Method of allocation of groups 8**

**1.4 Duration of the intervention period 8**

**1.5 Frequency and duration of follow-up 8**

**1.6 Visit windows 8**

**1.7 Data collection 8**
1.7.1 Eligibility screening 9
1.7.2 Primary outcome measure 9
1.7.3 Secondary outcome measures 9
1.7.4 Process indicators 10
1.7.5 Adverse events 11

**1.8 Sample size estimation (including clinical significance) 11**

**1.9 Brief description of proposed analyses and any pre-analysis statistical checks required 11**

*2. Data analysis plan – Data description 12*

**2.1 Recruitment and representativeness of recruited patients 12**

**2.2 Baseline comparability of randomised groups 14**

**2.3 Adherence to allocated treatment and treatment fidelity 14**

**2.4 Loss to follow-up and other missing data 14**

**2.5 Adverse event reporting 14**

**2.6 Assessment of outcome measures (unblinding) 14**

**2.7 Descriptive statistics for outcome measures 14**

**2.8 Description of intervention 15**

*3. Data analysis plan – Inferential analysis 15*

**3.1 Main analysis of treatment differences 15**
3.1.1 Analysis of primary outcome 15
3.1.2 Analysis of secondary outcomes 16
3.1.3 Statistical considerations 16
3.1.4 Sensitivity analyses 17
3.1.5 Planned subgroup analyses 18

**3.2 Exploratory analyses 18**

**3.3 Exploratory mediator and moderator analysis 18**

**3.4 Interim analysis 18**

*4. Software 18*

***B) SCHEDULE OF ASSESSMENTS AND MEASURES 19***

***Appendix 21***

**A1 Scoring guidelines ............ 21**

A1.1 Primary outcome ............ 21

A1.2 Secondary outcomes ............ 21

A1.3 Process evaluation parameters ............ 22

***Reference list 23***

## A) QUANTITATIVE ANALYSIS PLAN

**Investigators:** Dr Ben Carter
Prof Paul Stallard
Prof Jessica Deighton
Miss Jynna Yarrum
Dr Crispin Day
Dr James Shearer
Prof Sarah Byford
Dr Tim Weaver
Prof Peter Fonagy
Clare Evans

**Principal investigator:** Dr June Brown

**Trial manager:** Stephen Lisk

**Trial statisticians:** Kirsty James, Jodie Lord

## 1. Description of the trial

This trial aims to examine the clinical and cost-effectiveness of a multi-centre cluster RCT (cRCT) of DISCOVER for depression versus a control of usual school provision, in 60 schools over 4 sites.

### 1.1 Principal research objectives to be addressed

#### 1.1.1 Primary objective

To investigate the clinical effectiveness of DISCOVER on symptoms of depression in 16–18-year-olds over 6 months, assessed using the Mood and Feelings Questionnaire.

#### 1.1.2 Secondary objectives

1. To determine the feasibility of running a UK wide confirmatory trial of the DISCOVER intervention.
2. To assess the clinical effectiveness of DISCOVER on anxiety.
3. To assess the clinical effectiveness of DISCOVER on wellbeing.
4. To assess the clinical effectiveness of DISCOVER on sleep.
5. To assess the clinical effectiveness of DISCOVER on resilience.

6. To assess the cost-effectiveness of DISCOVER compared to control treatment in terms of quality adjusted life years (QALYs).
7. To descriptively assess the accessibility of the workshops for hard-to-reach populations (e.g., BME students, those who have not previously accessed NHS services or school counselling).
8. To assess the acceptability of the intervention when workshops are run by CWPs (Children and young people's Wellbeing Practitioner) or EMHPs (Educational Mental Health Practitioner).
9. To examine contextual (e.g., school) and process (e.g., workshop publicity) factors that influence implementation and clinical effectiveness.

### 1.2 Trial design including blinding

A two-arm blinded (researchers, statistician responsible for analysis (until SAP signoff, see below), senior statistician), UK wide multi-centre cluster randomised controlled trial (cRCT) with 3- and 6-month follow-up. The trial will be a clinical effectiveness and cost effectiveness evaluation and will take place in 60 secondary schools in 4 regions of the UK. Timings fit around the school year, within a 3-year timeframe, to enable recruitment over 2 school years.

The senior statistician BC will remain fully blind throughout the study. Trial statistician KJ was fully blind while drafting the version of this SAP that was circulated to the oversight committees and then became unblinded for the purpose of completing the internal pilot analysis. Trial statistician JL was fully blind until the SAP was signed off. In line with our statistical SOP on blinding ST-06 V3.1, the trial statistician may be unblinded once the SAP has been signed off.

***Figure 1. Trial design flow diagram***

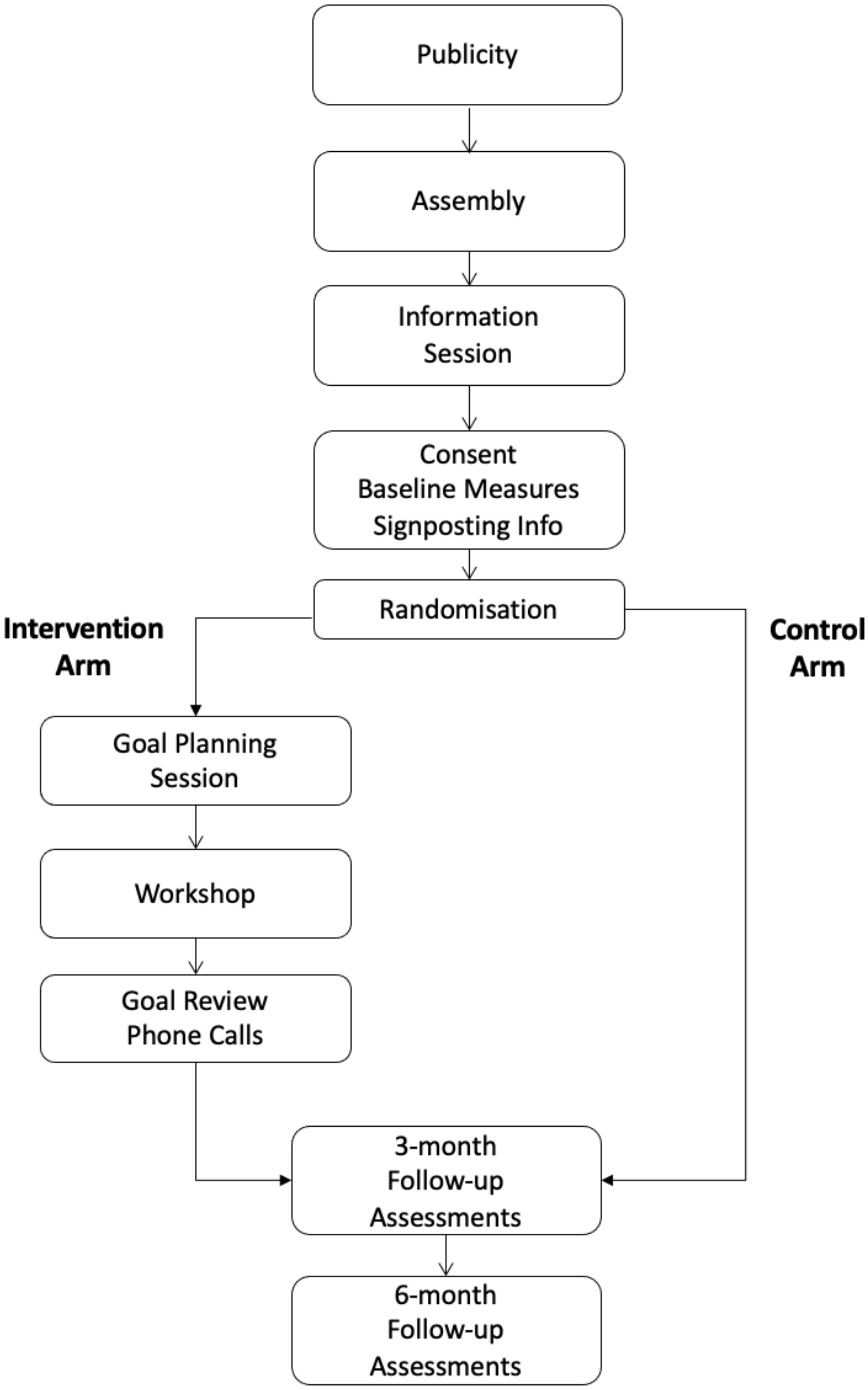

### 1.3 Method of allocation of groups

The randomisation allocation sequence will be generated at the level of the school. Baseline measures will be collected in the two weeks prior to randomisation. Once participant baseline assessments are complete, the clusters will be randomised to one of the treatment arms. Randomisation will be done in a 1:1 ratio. The partially blinded (A/B) randomisation sequence will be generated by the trial analyst/s and provided to the trial manager who will hold the randomisation key and will communicate with the schools to inform them of arm allocation and arrange intervention delivery. The sequence will be generated using a randomisation algorithm for cRCT developed by Carter [1], and will be stratified by Site (Bath, London, Manchester, Northampton), with balancing covariates consisting of school size and index of multiple deprivation. Cluster allocations will be sent to the trial manager once all participant baseline data has been collected from that cluster, the date that this allocation is sent will be considered as the date of randomisation for all follow up timepoints to be tied to. Workshops will take place in the month following randomisation.

### 1.4 Duration of the intervention period

The intervention will consist of a one-day workshop followed by up to 3 follow up phone calls which will happen within 3 months of randomisation.

### 1.5 Frequency and duration of follow-up

Participants will complete follow up measures at 3- and 6-months post randomisation.

### 1.6 Visit windows

Visit windows for 3 and 6 month follow up will be +/- 4 weeks for 3 months and +/- 6 weeks for 6 months.

### 1.7 Data collection

The research worker (RW) will meet with students individually to obtain informed consent and collect baseline data. Students will be contacted by text message prior to each assessment. The blinded RW will also conduct the follow up assessments.

The participants are initially contacted via the designated school staff to arrange follow up appointments. If they do not attend, a reschedule is attempted. If this appointment is missed the participants will be contacted directly by email (this is done using the central BESST email address) to schedule the 3- and 6-month follow-up assessments. However, should this fail, non-responders will be sent assessment questionnaires (either passed on to the student directly from school staff or sent via post by the school) along with instructions for completion, and asked to return these by post. No participant address information is held by the research team, instead the

team forward on CRFs to schools in sealed envelopes containing the student's name and a stamp and these are then forwarded on to the students by school staff.

### 1.7.1 Eligibility screening

Inclusion criteria (clusters)
- Secondary school with 6th form or dedicated 6th form college
- State-funded
- Sufficient resources available to host trial

Exclusion criteria (clusters)
- Further education college
- Privately funded school/college
- 6th form / College student population <70

Inclusion criteria (participants)
- Aged 16-18 years old.
- Planned attendance at the school or college until the end of the summer term.
- Sufficient English to provide valid informed consent and complete assessments in BESST study.
- Seeking help for stress, worry, or low mood (with or without teacher encouragement).
- Available to attend and take part in the workshop.

Exclusion criteria (participants)
- Identified as actively suicidal
- Current involvement with Child and Adolescent Mental Health Services for treatment of clinical mental health issues
- Existing diagnosis of severe learning difficulties or psychosis (as indicated on consent form)

### 1.7.2 Primary outcome measure

The primary efficacy parameter will be a measure of mood, using the Mood and Feelings Questionnaire (MFQ) - long version [2]. The MFQ is a 33-item self-reported depression measure, with good validity and reliability in adolescent samples. Scores range from 0-66, with a clinical cut-off of >27. This time-effective, validated, and standardised measure is the most suitable to assess symptom change.

### 1.7.3 Secondary outcome measures

- The Revised Child Anxiety and Depression Scale (RCADS) – child version [3] is a 47-item self-report measure, with good construct validity, internal consistency, and test-re-test reliability and has been used in older

adolescents up to the age of 18. Only the Anxiety Sub-scale will be used, and this will be presented as a t-score as per the scoring guidelines [3].

- The Warwick Edinburgh Mental Wellbeing scale (WEMWBS) [4] is a 14-item self-reported measure of mental well-being, successfully used with adolescents.
- Child and Adolescent Service Use Schedule (CA-SUS) [5]. We will use a version of the CA-SUS which has been successfully employed with young mental health populations, including school-based studies and depressed populations. The CA-SUS will be completed in interview with participants at baseline (for the previous month) and at 3 and 6-month follow-up (for the period since last interview). Data on the use of the DISCOVER intervention will be collected from the DISCOVER monitoring form. **(Not analysed by trial statistician outside of question 21 which will be used to inform a per-protocol analysis)**
- Sleep Condition Indicator [6]. The Sleep Condition Indicator (SCI) is a brief 8-item scale which measures sleep problems against the DSM-5 criteria for insomnia disorder. The SCI is valid, reliable, and sensitive to change.
- The Child and Youth Resilience Measure 12 (CYRM-12) [7]. The CYRM-12 is a 12-item scale designed as a screening tool to explore the resources (individual, relational, communal, and cultural) available to individuals, that may bolster their resilience.
- EQ-5D-3L [8]. The EQ-5D-3L is a self-report measure of health-related quality of life, used to calculate quality-adjusted life years (QALYs) suitable for economic evaluation. We are proposing the 3-level version over the EQ-5D-5L (5-level version) for several reasons: firstly, the recent position statement from NICE; secondly, the fact that the EQ-5D-3L version was tested in the feasibility study and found to be acceptable; and finally, there is evidence of the validity of the EQ-5D-3L for use with adolescent depression populations, which is not available for the EQ-5D-5L. **(Not analysed by trial statistician)**

### 1.7.4 Process indicators

- The Client Satisfaction Questionnaire [9] is an 8-item self-report measure of service satisfaction.
- Student Feedback on Workshops. A form will be co-developed with Young Advisors involved in DISCOVER PPI work which will involve:
  1. A feedback form completed at the end of the workshop, with open-ended questions about the workshop that were i) liked ii) disliked iii) most helpful and iv) could be improved. **(Not analysed by trial statistician)**
  2. Satisfaction with workshop at post-workshop using the CSQ.
  3. Techniques used in 3-month period following the interventions, recording during follow-up phone calls.
- The qualitative process evaluation is designed to (a) examine the contextual and process factors that either support or obstruct the implementation of the intervention, (b) examine the experience of participants and workshop

facilitators and (c) assess whether and how the contextual (e.g. school regime and environment) and process factors (e.g. publicity, component parts of the workshop) identified through this work influence the intermediary outcomes (e.g. engagement, intervention fidelity, adherence to intervention protocol) as well as the primary and secondary outcomes assessed in the trial. The qualitative process evaluation will be conducted in 8 intervention schools using semi-structured interviews (with n=16 students, n=8 workshop facilitators) and focus groups with school staff (n=8). **(Not analysed by trial statistician)**

#### 1.7.5 Adverse events

Research workers from each site will note AEs/SAEs at each follow up interview and enter these into the MACRO database. Any SAEs or suspected SAEs that are recorded will be reported to the Trial Manager. Facilitators will record AEs/SAEs from the workshop and follow-up calls, all these events will be reported to the trial manager to enter onto the trial database. All AEs/SAEs will be summarised and reported in the Open report of the Data Monitoring Committee (DMC) which will also be circulated to the Trial Steering Committee (TSC). SAEs will also be circulated to the DMC chair for review. Action will then be taken accordingly depending on implications for the conduct of the trial.

### 1.8 Sample size estimation (including clinical significance)

Based on the feasibility study [10], where estimated ICCs (intraclass correlations) were found to be negligible (between 0 and 0.003), we estimate that to detect a mean change score of 5.6 with a two-sided alpha of 0.05 on the Mood and Feelings Questionnaire (MFQ) in the intervention group and 2.8 in the Control group with pooled SD=10 (Effect size 0.28, 90% power), we need an ICC of 0.02. However, we have increased the ICC from 0.02 to 0.03 which is consistent with typical ICC found from other studies of mental health interventions of mood outcomes found in schools in the UK [11]. This has increased the number of schools required from 54 to 60, increasing the number of students from 810 to 900, with 15 students per school (average). This will give 90% power to detect the pre-specified target difference for the primary outcome. This assumes a loss to follow up of 12.5% of students, and a further 4% (N=2) schools dropping out, based on the low dropout rate from the feasibility study.

### 1.9 Brief description of proposed analyses and any pre-analysis statistical checks required

Prior to database lock the statistician will perform data checks and generate queries for review by those responsible for entering data on the following:

- Ensuring that the CONSORT diagram can be completed with agreement between status forms/withdrawal forms and available data

- Ensuring that all available primary and secondary outcome data has been entered by providing lists of pins of participants who do not have each measure entered
- Checking inconsistencies between data entered in the trial database and data extracted from the randomisation system
- Missing baseline data

Analyses will be carried out by the trial statistician and overseen by BC. The trial statistician who is responsible for performing the analysis (JL) will remain blind to treatment allocation until the SAP has been finalised and approved, BC will remain fully blind until all analysis has been completed.
The Primary and Secondary analyses will use the modified ITT population. This will be modified to include all participants that provide at least one post baseline outcome assessment of interest. This population will be referred to as the ITT population. Health economic outcomes, CA-SUS and EQ-5D-3L will be analysed by the health economists and so will not be detailed here. Qualitative analysis will also not be undertaken by the trial statistician so is not detailed here.

## 2. Data analysis plan – Data description

### 2.1 Recruitment and representativeness of recruited patients

CONSORT flow chart will be constructed (12) – see Figure 2. This will include the number of eligible students (n) and clusters (m), number of students and clusters agreeing to enter the trial, number of students and clusters refusing, then by treatment arm: the number of students continuing through the trial, the number of students and clusters withdrawing from the trial, and the number of students lost to follow-up.

*Figure 2.  Template CONSORT diagram for BESST trial*

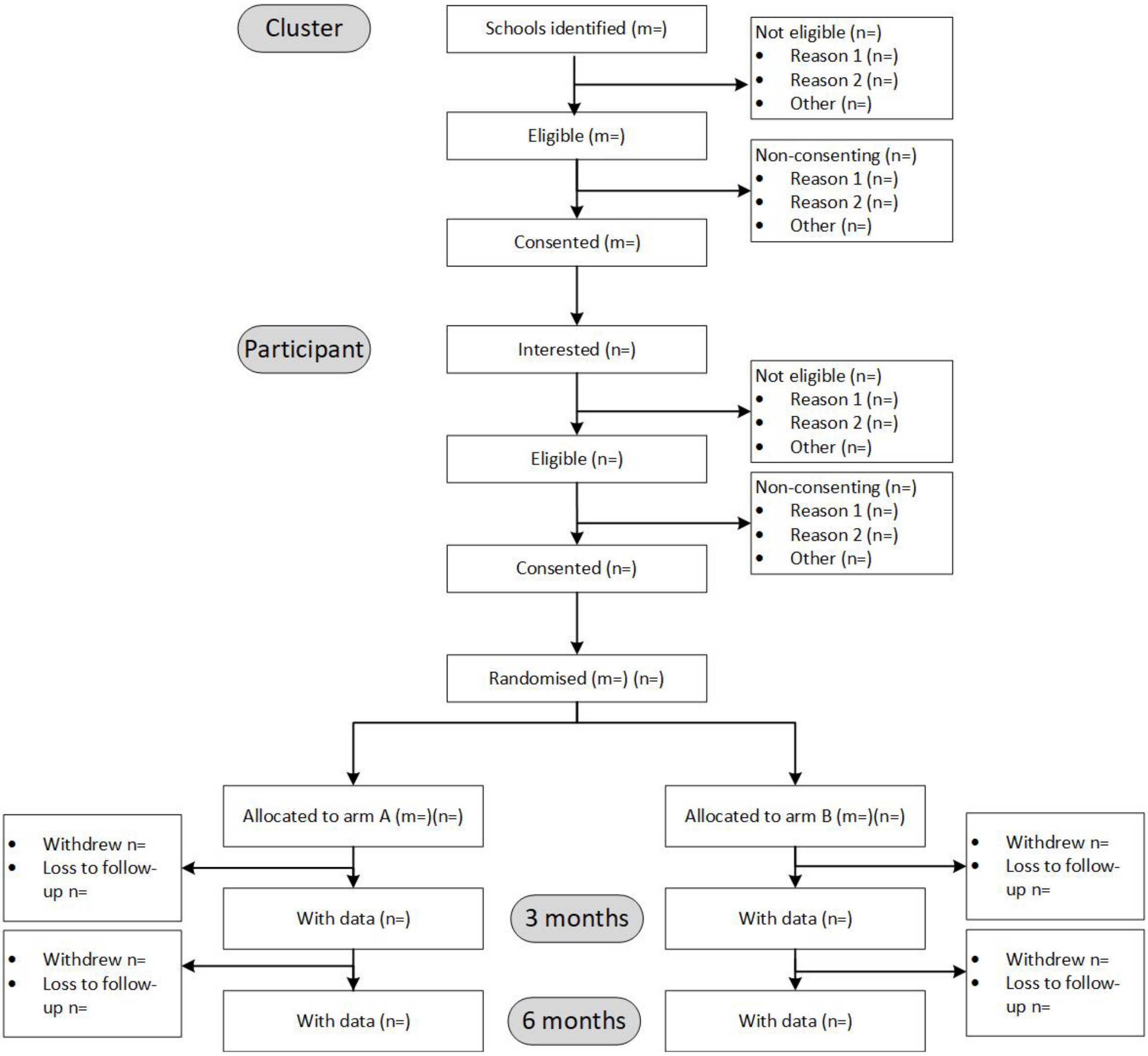

## 2.2 Baseline comparability of randomised groups

Baseline descriptions of clusters and participants by arm and overall will be presented as means and standard deviation or numbers and proportions as appropriate. We will not perform any significance tests to look at differences between the groups at baseline as any difference will be due to chance.

## 2.3 Adherence to allocated treatment and treatment fidelity

Treatment adherence to the workshop and take up of optional follow up calls will be collected in the intervention log and will be summarised. We will summarise reasons for participants not attending the full workshop and the content and length of the optional goal review follow up calls.

Fidelity of the intervention will be assessed in the qualitative study and so is not described in detail here.

## 2.4 Loss to follow-up and other missing data

The number of participants with missing data for each outcome will be summarised in each arm and at each time point. The number withdrawing from treatment and reasons for withdrawal from treatment will be summarised by treatment group and overall. For those who withdraw from treatment only, follow-up data will still be collected where possible. The frequency and proportion of people consented who withdraw from the trial will be presented. If any cluster withdraws from the trial, reasons will be summarised, and we will continue to collect participant follow up data where possible. If a participant withdraws from the trial, then no further follow up data will be collected.

## 2.5 Adverse event reporting

Research workers from each site will note AEs/SAEs at each follow up interview and enter these into the MACRO database. Any SAEs or suspected SAEs that are recorded will be reported to the Trial Manager. Facilitators will record AEs/SAEs from the workshop and follow-up calls, all these events will be reported to the trial manager to enter onto the trial database. All AEs/SAEs will be summarised by arm and overall, by type, relationship to study and severity. We will summarise number of events and number of students who have experienced events.

## 2.6 Assessment of outcome measures (unblinding)

Outcome assessors will be blind to allocation. Measures that are collected in one arm only (for example, goal planning and client satisfaction questionnaires) will be collected by an unblinded assessor.

## 2.7 Descriptive statistics for outcome measures

The primary and secondary measures will be summarised using appropriate summary statistics, for the entire trial sample and by trial group at each time point.

The distributions of the continuous outcome measures will be inspected, and a judgement made on whether the variables are normally distributed or not. The mean and standard deviation will be presented for all normally distributed measures, median and quartiles for skewed distributions and proportions and frequencies for categorical measures. The primary and secondary outcomes mean, and confidence intervals will be plotted over time in an unadjusted mean profile plot. Ranges will also be reported for each of the outcome measures.

### 2.8 Description of intervention

The intervention will be summarised using the process indicator measures looking at satisfaction of the service (CSQ) and feedback from the participants on the workshop including open ended questions about what they liked, disliked, found most helpful and what they thought could be improved.

## 3. Data analysis plan – Inferential analysis

### 3.1 Main analysis of treatment differences

The primary clinical question of interest (estimand) is what is the difference in depressive symptoms (measured by the MFQ) for 16-18 year olds (as defined by the trial inclusion/exclusion criteria) at 6 months follow up when comparing TAU to the DISCOVER workshop, regardless of discontinuation of study treatment. Mean group difference estimates and associated confidence intervals will be reported.

The estimand is defined using the following attributes:

- **Population**: 16–18-year-old students from a randomised school who self-identify as needing help with stress and meet the trial inclusion/exclusion criteria.
- **Endpoint**: group difference in depressive symptoms will be compared at 6 months post randomisation
- **Treatment condition:** DISCOVER compared to TAU regardless of discontinuation for any reason (treatment policy strategy)
- **Remaining intercurrent events**: discontinuation is already addressed through the choice of the treatment policy strategy. We will explore the pattern of withdrawals to ensure follow up data is missing at random and similar by arm. If not, remedial action will be taken which will be drafted by a fully blinded analyst (BC) and approved by the TSC independent statistician. There are no other intercurrent events of note.
- **Population-level summary**: the mean MFQ for each group.

**Rationale for estimand:** we want to compare DISCOVER to TAU as would be observed in routine practice.

#### 3.1.1 Analysis of primary outcome

The main analysis will include all randomised participants who provide follow up date from at least one timepoint as per randomisation, irrespective of treatment

received post randomisation and irrespective of whether they fall inside or outside of collection windows. This ITT population will be used to estimate the treatment policy based primary estimand.

MFQ scores will be analysed using a mixed-effect multilevel linear model adjusted by the following fixed effects: aggregated level school deprivation; geographical area; school size; gender; BME group; a dummy variable indicating treatment group, and baseline severity (MFQ score). A treatment group by time interaction term will be including to allow for extracting comparisons at both follow up times. A random intercept will be fitted for each school, and student, and the difference between the intervention and control MFQ score will be estimated, alongside the 95% confidence interval and p-value. We will also calculate the overall ICC and 95% CIs of the primary outcome at 3 and 6 months follow up.

### 3.1.2 Analysis of secondary outcomes

Continuous secondary outcomes will be analysed in the same way as the primary outcome. Adverse events will be analysed as a binary outcome coded as 1 for participants with any events occurring and 0 for those with no events. This analysis will also be repeated with respect to severity, 1= any severe event occurred 0= no serious events occurred.

Process indicator scales that are collected in the intervention arm only will be summarised with means and standard deviations or medians and inter-quartile ranges as appropriate. Only the CSQ-8 and the techniques used will be summarised by the trial statistician.

### 3.1.3 Statistical considerations

**Time points**

All measures will be recorded on dedicated sessions at pre-intervention, and 3- and 6-month follow-up time points. The measures will be collected via individual interviews between the blinded research worker and each participant.

**Stratification and clustering**

All stratification variables used in the randomisation algorithm will be included in the analysis models. We will also include a random intercept for school and participant to allow for clustering within school and between outcome measures collected at different timepoint for each participant.

**Missing items in scales and subscales**

The number (%) of participants with complete data will be reported. The ideal approach would be to use missing value guidance provided for scales. As an alternative, where no guidance is available, scales will be pro-rated for an individual if 20% or fewer items are missing. For example, in a scale with 10 items, prorating will be applied to individuals with 1 or 2 items missing. The average value for the 8 or 9 complete items will be calculated for that individual and used to replace the missing values. The scale score will be calculated based on the complete values and these replacements.

**Missing baseline data**

Missing baseline data should not be an issue for the primary analysis. If these do contain missing data, the number with complete data will be reported and they will be imputed using a method suitable to the variable as per the recommendations of White and Thompson (13).

**Missing outcome data**

Where there are two or more outcome time points, missing post-randomisation assessments will be dealt with by fitting linear mixed models to all the available data using maximum likelihood methods. Such an approach provides valid inferences under the assumption that the missing data mechanism is ignorable (or MAR).

**Method for handling multiple comparisons**

As we have a single prespecified primary outcome, all secondary analyses can be considered as exploratory therefore adjusting for multiple comparisons is not necessary.

**Method for handling non-compliance (per protocol/CACE analyses)**

In addition to the primary intention-to-treat analysis, the effect of actually receiving treatment as defined in the protocol will also be estimated. An acceptable dose of intervention will be considered as participant attendance of 75% or over of the workshop and completion of goal setting.

For the per protocol analysis, we will exclude those who meet the following criteria:

1. Participants who have not received an acceptable dose of the intervention.
2. Participants with data collected outside visit windows
3. Participants who have accessed CAMHS (question 21 of CAS-US) since baseline

If non-compliance with the active treatment is high (>40%), a CACE analysis will be considered.

**Model assumption checks**

The continuous models assume normally distributed residuals. Linear mixed model residuals will be plotted to check for normality and inspected for outliers. If the distribution of the residuals deviates substantially from normal, we will fit the models requesting robust standard errors. Any issues that arise with model assumptions that lead to a change of the planned analysis will be reviewed by the blinded senior statistician and approved by the blinded independent TSC statistician.

### 3.1.4 Sensitivity analyses

We will repeat the primary analysis including just the participants with a score of greater than 27 on the MFQ at baseline.

#### 3.1.5 Planned subgroup analyses

We will repeat the primary outcome analysis looking at male and female participants separately.

We will also repeat the primary outcome analysis looking at participants in their first year of study and those in their second year of study separately.

### 3.2 Exploratory analyses

None

### 3.3 Exploratory mediator and moderator analysis

None

#### 3.4 Interim analysis

No formal interim analysis of the primary or secondary outcomes is planned. However, after completion of year 1, we will assess the feasibility of continuing the BESST trial, presenting the findings of an internal pilot to the DMC. We will compare our recruitment, retention, and fidelity, and only progress to the full trial by meeting the following 'Go'/'No go' criteria, as indicated by meeting the green criteria. Less than this value will result in being flagged as amber and or red, as per the boundaries listed in the following table.

| | Red* | Amber | Green |
|---|---|---|---|
| Randomising 19 schools | <15 | 15 to 18 | ≥19 |
| 3- and 6-month follow-up measures conducted in 80% of randomised schools | <12 | 12 to 15 | ≥15 |
| Recruit 180 students to the trial | <144 | 144 to 179 | ≥180 |
| Participant adherence (% of students followed up) | <64% | 64 to 79% | ≥80% |
| 60% of students from the intervention arm will give satisfaction ratings on the CSQ of at least 26 points | <48% | 48 to 59% | ≥60% |

## 4. Software

Data management: An online data collection system for clinical trials (MACRO; InferMed Ltd) will be used to enter and store data which has been collected for the study across all timepoints. This is hosted on a dedicated server at KCL and managed by the KCTU. The KCTU Data Manager will extract data periodically as needed and provide these in Stata format to the analyst(s). No other member of the trial team should be requesting data extracts, particularly any extracts containing outcome data.

Statistical analysis: Stata 17 will be used for data description and the main inferential analysis.

### *B) SCHEDULE OF ASSESSMENTS AND MEASURES*

| | Publicity at school | Study Present -ation | RW meets Students & pre-measures | Pre-workshop goal planning meetings | Intervention | Post-intervention measures | Up to 3 Follow-Up Calls | 3-month follow-up measures | 6-month follow-up measures | Interviews |
|---|---|---|---|---|---|---|---|---|---|---|
| 6th form assembly | X | | | | | | | | | |
| Lunchtime information meeting | | X | | | | | | | | |
| Consent | | | X | | | | | | | |
| Socio-demographics | | | X | | | | | | | |
| Referral Route (teacher assisted/not) | | | X | | | | | | | |
| Ask Suicide-screening Questions (ASQ) | | | X | | | | | | | |
| Mood & Feelings Questionnaire (MFQ) | | | X | | | | | X | X | |
| Revised Child Anxiety & Depression Scale | | | X | | | | | X | X | |
| Warwick Edinburgh Mental Wellbeing Scale | | | X | | | | | X | X | |
| Sleep Condition Indicator | | | X | | | | | X | X | |
| Child and Youth Resilience Measure | | | X | | | | | X | X | |
| Student Feedback Form | | | | | | X (Exp arm only) | | | | |
| Client Satisfaction Questionnaire | | | | | | X (Exp arm only) | | | | |
| Techniques | | | | | | | X (Exp | | | |

| | | | | | | | | | | |
|---|---|---|---|---|---|---|---|---|---|---|
| Used | | | | | | | arm only) | | | |
| Child & Adolescent Service Use Schedule (CA-SUS) | | | X | | | | | X | X | |
| EQ-5D-3L | | | X | | | | | X | X | |
| Randomisation of Schools | | | | | | | | | | |
| Intervention | | | | | X (for schools in Exp arm only) | | | | | |
| Goal Planning | | | | X (Exp arm only) | X (Exp arm only) | | X (Exp arm only) | | | |
| Process Evaluation | | | | | | | | | | X |

## Appendix

## A1 Scoring guidelines

### A1.1 Primary outcome

**MFQ** is collected at baseline, 3- and 6- month follow-up with the highest possible score being 66.

Total is obtained by summing all 33 items with no reverse scoring, the higher the score the more severe the condition.

No guidelines provided for missing data, a conservative approach of standard prorating if 20% or less missing values will be used.

### A1.2 Secondary outcomes

**RCADS Total anxiety score** is collected at baseline, 3- and 6- month follow-up, we will use the total raw score which has the highest possible score of 111. Item scores range from 0=Never to 3=always

Subscale items:
Social Phobia: 4, 7, 8, 12, 20, 30, 32, 38, 43
Panic Disorder: 3, 14, 24, 26, 28, 34, 36, 39, 41
Major Depression: 2, 6, 11, 15, 19, 21, 25, 29, 40, 47
Separation Anxiety: 5, 9, 17, 18, 33, 45, 46
Generalized Anxiety: 1, 13, 22, 27, 35, 37
Obsessive-Compulsive: 10,16, 23, 31, 42, 44

Total anxiety score is obtained by summing all the subscales apart from the major depression subscale with no reverse scoring, the higher the score the more severe the condition.

Missing data for raw scores can be handled by prorating the remaining items within a scale. It is recommended that scales with more than 2 missing items are not scored. Likewise, the total anxiety score can have up to 10 missing items, but only if each subscale has no more than 2 missing; and the total anxiety and depression score can have up to 12 missing items, but only if each subscale has no more than 2 missing items. To estimate the scale score, take the sum of the completed items within that scale and divide that by the number of items completed, then multiple by the total number of items in that scale, and then round the result. For example, if one item is missing from the separation anxiety scale (which has seven items), and the 6 completed items sum to 4, you would divide 4 by 6 (0.67), and then multiply by 7, which would yield 4.67, which then rounds to 5. Thus, you would count the score as a 5 not a 4 because of the prorating.

Raw scores will then be converted to t-scores as per the scoring guidelines.

**WEMWBS** is collected at baseline, 3- and 6- month follow-up with the highest possible score being 70.

Total is obtained by summing all 14 items with no reverse scoring, the higher the score the less severe the condition.

No guidelines provided for missing data, a conservative approach of standard prorating if 20% or less missing items will be used.

**SCI** is collected at baseline, 3- and 6- month follow-up with the highest possible score being 32.

Total is obtained by summing all 8 items with no reverse scoring, the higher the score the less severe the condition.

No guidelines provided for missing data, a conservative approach of standard prorating if 20% or less missing items will be used.

**CYRM-12** is collected at baseline, 3- and 6- month follow-up with the highest possible score being 60.

Total is obtained by summing all 12 items with no reverse scoring, the higher the score the higher the resilience.

No guidelines provided for missing data, a conservative approach of standard prorating if 20% or less missing items will be used.

### A1.3 Process evaluation parameters

**CSQ-8** is collected at baseline, 3- and 6- month follow-up with the highest possible score being 32.

Total is obtained by summing all 8 items with no reverse scoring, the higher the score the higher the satisfaction.

Guidelines provided for missing data suggest prorating if 4 or more questions are answered.

**Reference list**


1. Carter BR, Hood K: Balance algorithm for cluster randomizedrandomised trials. *BMC Medical Research Methodology* 2008, 8(1):65.
2. Costello E, Angold A: Scales to assess child and adolescent depression: checklists, screens and nets. *Journal of the American Academy of Child & Adolescent Psychiatry* 1988, 27(6):726-737.
3. Chorpita BY, L.; Moffitt, C.; Umemoto, LA.; Francis, SE: Assessment of symptoms of DSM-IV anxiety and depression in children: A revised child anxiety and depression scale. *Behaviour Research and Therapy* 2000, 38(8):835-855
4. Tennant R, Hiller L, Fishwick R, Platt S, Joseph S, al WSe: The Warwick-Edinburgh Mental Well-being scale (WEMWBS): development and UK validation. *Health and Quality of Life Outcomes* 2007, 5:63.
5. Ford T, Hayes R, Byford S, Edwards V, Fletcher M, Logan S, Norwich B, Pritchard W, Allen K, Allwood M *et al*: The effectiveness and cost-effectiveness of the Incredible Years® Teacher Classroom Management programme in primary school children: results of the STARS cluster randomised controlled trial. . *Psychological Medicine* 2012, 12:719.
6. Espie, C. A., Kyle, S. D., Hames, P., Gardani, M., Fleming, L., & Cape, J. (2014). The Sleep Condition Indicator: a clinical screening tool to evaluate insomnia disorder. BMJ open, 4(3)
7. Liebenberg, L., Ungar, M., & LeBlanc, J. C. (2013). The CYRM-12: a brief measure of resilience. Canadian Journal of Public Health, 104(2), e131-e135.
8. Brooks R: EuroQol: the current state of play. *Health Policy* 1996, 37(1):53-72
9. Larsen DL, Attkisson C, Hargreaves WA, Nguyen TD: Assessment of client/patient satisfaction: development of a general scale. *Evaluation and Program Planning* 1979, 2:197-207.
10. Brown JSL, Blackshaw E, Stahl D, Fennelly L, McKeague L, Sclare I, Michelson D: School-based early intervention for anxiety and depression in older adolescents: A feasibility randomised controlled trial of a self-referral stress management workshop programme (“DISCOVER”)
11. Shackleton N, Hale D, Bonell C, Viner RM: Intraclass correlation values for adolescent health outcomes in secondary schools in 21 European countries. SSM - Population Health 2016, 2:217-225.
12. Campbell, M. K., Elbourne, D. R., & Altman, D. G. (2004). CONSORT statement: extension to cluster randomised trials. Bmj, 328(7441), 702-708.
13. White IR, Thompson SG. Adjusting for partially missing baseline measurements in randomized trials. Stat Med 2005 Apr 15;24(7):993-1007.
